# Estimation of Isentropic Compressibility of Biodiesel Using ELM Strategy: Application in Biofuel Production Processes

**DOI:** 10.1155/2021/7332776

**Published:** 2021-07-12

**Authors:** Marischa Elveny, Meysam Hosseini, Tzu-Chia Chen, Adedoyin Isola Lawal, S. M. Alizadeh

**Affiliations:** ^1^Data Science & Computational Intelligence Research Group, Universitas Sumatera Utara, Medan, Indonesia; ^2^Department of Mathematics, Campus of Bijar, University of Kurdistan, Sanandaj, Kurdistan, Iran; ^3^CAIC, DPU, Bangkok, Thailand; ^4^Dept. of Accounting and Finance, Landmark University, Omu-Aran, Nigeria; ^5^Sustainable Development Goal 17 (Partnership for the Goals) Research Cluster, Landmark University, Nigeria; ^6^SDG 8 (Decent Work and Economic Growth) Research Cluster, Landmark University, Nigeria; ^7^SDG1 (Zero Hunger) Research Cluster, Landmark University, Nigeria; ^8^SDG6 (Clean Energy) Research Cluster, Landmark University, Nigeria; ^9^Petroleum Engineering Department, Australian College of Kuwait, West Mishref, Kuwait

## Abstract

Isentropic compressibility is one of the significant properties of biofuel. On the other hand, the complexity related to the experimental procedure makes the detection process of this parameter time-consuming and hard. Thus, we propose a new Machine Learning (ML) method based on Extreme Learning Machine (ELM) to model this important value. A real database containing 483 actual datasets is compared with the outputs predicted by the ELM model. The results of this comparison show that this ML method, with a mean relative error of 0.19 and *R*^2^ values of 1, has a great performance in calculations related to the biodiesel field. In addition, sensitivity analysis exhibits that the most efficient parameter of input variables is the normal melting point to determine isentropic compressibility.

## 1. Introduction

The suitability of oils and fats is often determined by their physicochemical properties. The fact that the terms “oil” and “fat” are used interchangeably in many languages indicates that the liquid or solid state of products at room temperature is considered crucial in distinguishing these two classes of goods [1]. From a technical point of view, to design rational lipidic materials, a thorough understanding of the rheological behavior, molecular structure, crystallization, and melting characteristics of oils and fats is needed [2]. It is well understood that the properties of oils and fats are principally determined by their triacylglycerol (TAG) structure, which includes the degree of unsaturation and the carbon chain length of the fatty acid molecules in the TAG component [3–5].

Besides their importance in food processing, animal fats and vegetable oils have been viewed as important sustainable resources for biodiesel production due to the impending depletion of fossil fuels [6–8]. The wide range of source oil FA profile is linked to many important biodiesel parameters including density, pour point, cloud point, cold filter plugging point, and viscosity. As a result, animal or plant/seed sources utilized in biofuel production play an important role in biodiesel quality [9–11].

The laboratory assessment of physicochemical characteristics of oils and fats, like viscosity, density, composition, crystallization, and melting, necessitates the use of a variety of analytical tools, including differential scanning calorimeter, nuclear magnetic resonance, high-performance gas or liquid chromatography, and spectrometer analyzers [12–14]. Nevertheless, given the variety of feedstock, FA profiles, and lipids, this huge need for analytical instruments can make the physical characterization of these items an expensive and delaying process [15–17]. Since it is not possible to collect data on properties under all possible conditions, accurate methods for predicting them can be very useful for the design of products and processes [18]. In predictive modeling, the physicochemical phenomena-based models can be more complete and less constrained compared to simple polynomial or linear-fitted equations [19–22].

In general, the construction of models with a physics base begins with an understanding of the mathematics of the phenomena under investigation and then continues with doing simplifications to obtain a realistic model that presents a reasonable explanation for the phenomenon [23]. This analytical technique of modeling has commonly been applied in many fields, as shown by many literature references [24–26]. Although modeling approaches have primarily been utilized to explain thermodynamic features of fat and oil melting and crystallization, they have also been used in the production processes of biodiesel, process optimizations, and quantitative determination of biodiesel properties and compare them with thermodynamic characteristics [27, 28].

Given the above, a piece of detailed knowledge about the constraints and potentials of estimating modeling used in the measurement of physicochemical properties of biodiesel fuels is needed to fully exploit the opportunities presented by this method for the production of novel fat-based products and the processes of biodiesel production. In this paper, for the first time, the ELM algorithm is used to model and predict isentropic compressibility, one of the important properties of biodiesel. In this research work, after stating how to collect and use experimental data, the modeling method of this theorem is stated and in Results and Discussion, various analyses are used to evaluate the accuracy of this method.

## 2. Actual Data Collection

The database, including 483 data, related to this study was gathered from the literature [29]. In the following, we develop thorough the precise methods to estimate the isentropic compressibility. Also, variables were selected on the basis of existing data (including pressure, temperature, molecular weight, and melting point) due to having a predictive tool to estimate output values. It is noted that this database is divided into 120 testing data and 363 training data by chance. Then, after implementation of data, they are normalized as follows:

(1) $$\begin{array}{c} X_{N}=2\frac{x-x_{\min}}{x_{\max}-x_{\min}}-1. \end{array}$$

## 3. Extreme Learning Machine (ELM)

ELM is invented by Huang et al. that has a structure like a single-layer feed-forward Neural Network (NN). But they differ from each other because of lacking bias of the output neuron (ON) [30, 31]. In an ELM algorithm, every input layer neuron is linked to all of the neurons in the hidden layer (HL). So, all of the neurons in the HL can have values related to their own bias, and the activation function of the output layer has a linear form, while the activation function of the HL is in the form of a piecewise continuous function [32]. Unlike other algorithms such as the back-propagation algorithm or conjugate gradient descent, ELM uses another way to find bias and weight [33]. In this way, ELM uses an algorithm to determine weights and biases of input layer neurons and those of HL neurons, randomly. So, it is assumed that an ELM algorithm has “*i*” input neurons and “*k*” training cases with “*j*” HL neurons where the HL activation can be defined as follows:

(2) $$\begin{array}{c} H_{jk}=g\left(\sum \left(w_{ji}x_{ik}\right)+B_{j}\right), \end{array}$$

in which *H*_*jk*_ is the activation matrix of *j*_th_ HL neuron for the *k*_th_ training case, *g* is the nonlinear activation function, *B*_*j*_ is the bias of *j*_th_ HL neuron, *x*_*ik*_ is the *i*_th_ input neuron for *k*_th_ training neuron, and *w*_*ji*_ is the weight between *i*_th_ input neuron and *j*_th_ HL neuron.

The *i* × *j*-dimension *H* matrix shows all HL neurons activated for all training cases [34].

By fitting least-squares on targets in training, we can compute the weight values between the HL neurons and ON. This implementation is linear and performed by Eqs. (3)–(5) as follows [35, 36]:

(3) $$\begin{array}{c} H_{k\times j}\beta_{j\times 1}=T_{k\times 1}, \end{array}$$

(4) $$\begin{array}{c} \beta ={\left(\beta_{1}\cdots \beta_{j}\right)}_{j\times 1}, \end{array}$$

(5) $$\begin{array}{c} T={\left(T_{1}\cdots T_{k}\right)}_{k\times 1}, \end{array}$$

where *T* and *β* are the target vector of training cases and the weight vector of hidden neurons and ON. Rather than these equations, we can multiply the Moore-Penrose pseudoinverse matrix, *H*′, by *T*. This computation seems like the least-squares multilinear regression is implementing [37].

(6) $$\begin{array}{c} \beta =H^{\prime }T, \end{array}$$

in which, *H*′ is the inverse matrix, known as Moore-Penrose pseudoinverse of *H*. So, after doing these calculations, the network training is completed [38, 39].

In total, this training process has only two essential steps: (1) determination of random biases and weights for hidden neurons to find the HL output and (2) estimation of output weights using Moore-Penrose pseudoinverse of *H*. As mentioned before, the training process of the ELM algorithm is implemented by the determination of *H*′ for the HL. This process is much faster than other training methods such as Levenberg-Marquardt. This method does not utilize the approach of nonlinear optimization, and it is only dependent on a closed-form solution [40, 41].

## 4. Results and Discussion

In the following, some formulations are used to estimate various types of statistical indices for the ELM algorithm.

(7) $$\begin{array}{c} \text{Mean squared error }\left(\text{MSE}\right)=\frac{1}{N}\overset{N}{\underset{i=1}{\sum }}{\left(x_{I}^{\text{actual}}-x_{I}^{\text{predicted}}\right)}^{2}, \end{array}$$

(8) $$\begin{array}{c} \text{Mean relative error }\left(\text{MRE}\right)=\frac{100}{N}\overset{N}{\underset{i=1}{\sum }}\left(\frac{X_{I}^{\text{actual}}-X_{I}^{\text{predicted}}}{X_{I}^{\text{actual}}}\right), \end{array}$$

(9) $$\begin{array}{c} R-\text{squared }\left(R^{2}\right)=1-\frac{\sum_{i=1}^{N}\;{\left(x_{I}^{\text{actual}}-x_{I}^{\text{predicted}}\right)}^{2}}{\sum_{i=1}^{N}\;{\left(x_{I}^{\text{actual}}-\bar{x_{I}^{\text{actual}}}\right)}^{2}}, \end{array}$$

(10) $$\begin{array}{c} \text{Standard deviations }\left(\text{STD}\right)={\left(\frac{1}{N-1}\overset{N}{\underset{i=1}{\sum }}\left(\text{error}-\bar{\text{error}}\right)\right)}^{0.5}, \end{array}$$

(11) $$\begin{array}{c} \text{Root mean square error }\left(\text{RMSE}\right)=\sqrt{\frac{1}{N}\overset{N}{\underset{i=1}{\sum }}\left({\left(x_{I}^{\text{actual}}-x_{I}^{\text{predicted}}\right)}^{2}\right)}. \end{array}$$

Table 1 shows the various statistical analyses for the evaluation of the ELM model in predicting actual output values. As can be seen from this table, this model shows a high ability to predict output values. By comparing the coefficient of determination values obtained from this model with similar work done by Abooali et al. [29], the better performance of the proposed model can be concluded. They used SGB and GP models to predict isentropic compressibility values, and their models were able to estimate this parameter with coefficients of determinations 0.99993 and 0.99608, respectively.

**Figure 1:**
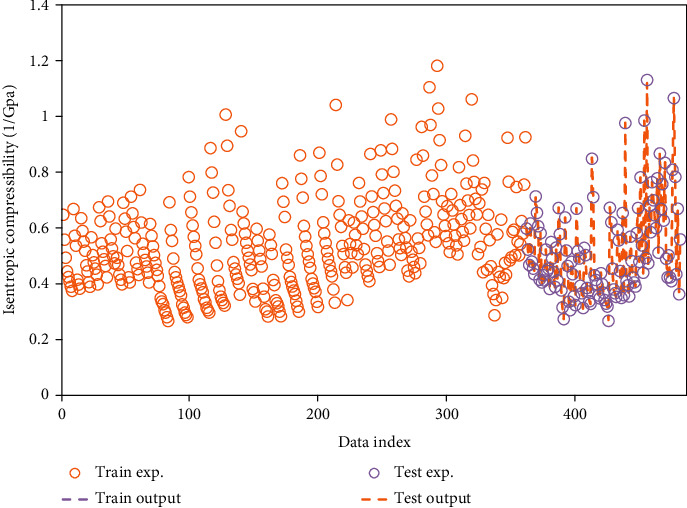
Simultaneous and visual comparison of real and corresponding modeled data in test and training phases.

**Figure 2:**
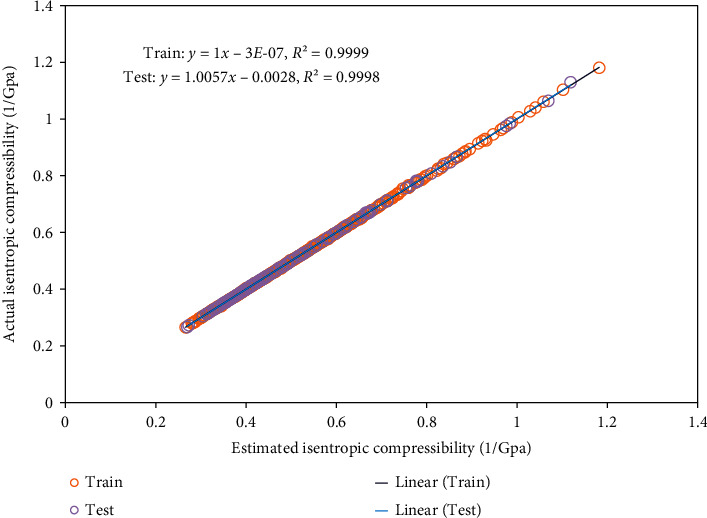
Cross plot analysis on the model to determine its accuracy in predicting actual values.

The comparison between modeled outputs and actual data is performed visually in Figure 1 that creates a helpful viewpoint about the precision of the ELM algorithm to make a prediction from the isentropic compressibility. Also, Figure 2 shows the cross plot of the regression of actual and modeled values of target data using the ELM algorithm. This prediction has an excellent agreement with real data for the model. As can be seen in this figure, the coefficients of determination related to the training and testing phases are 0.9999 and 0.9998, respectively.

**Figure 3:**
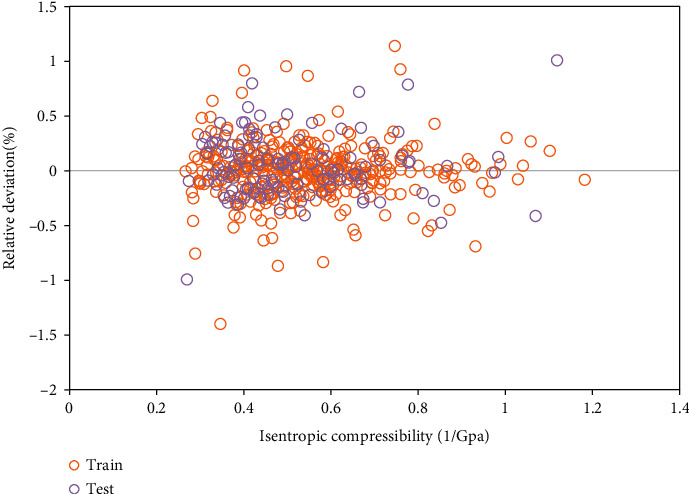
Relative deviation analysis on the model to determine its accuracy.

According to actual data, the assessment is done by the relative deviations of generated outcomes of the ELM algorithm that is shown in Figure 3. So, it is concluded that relative errors of this algorithm, resulted in the prediction of target values, are close to zero. Also, all of the relative error values of the ELM algorithm are less than 1.5%, which verifies its power.

It is noted that the database utilized for the preparation of the proposed model can affect the accuracy and reliability of this model [42]. One of the important steps to propose models with great accuracy is finding and removing suspected data. These data are the points that behave differently from others. So, to detect these kinds of data, Leverage analysis is employed. Also, to define the standardized residuals versus hat values, William's plot is used to recognize the outlying points. To determine the hat value on the basis of diagonal elements, the hat matrix is given as follows [43]:

(12) $$\begin{array}{c} H=A{\left(A^{T}A\right)}^{-1}A^{T}, \end{array}$$

where *A* is a *a* × *b*-dimension matrix which *a* and *b* are the number of the model parameter as well as training points, respectively. The squared limited area, known as reliable area, is enclosed by cut-off and warning leverage values of vertical and horizontal axes, respectively. The warning leverage values are calculated as follows:

(13) $$\begin{array}{c} H^{*}=\frac{3\left(b+1\right)}{a}. \end{array}$$

**Figure 4:**
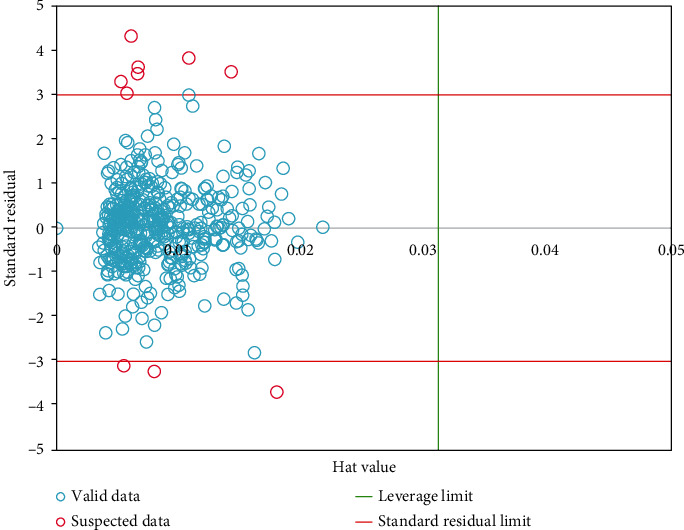
Leverage analysis to identify suspicious data.

Also, the cut-off value can be +3 and -3. The outlier detection of the ELM has been shown in Figure 4. As you see, the main number of the target data is placed within the nonsuspected/reliable area.

**Figure 5:**
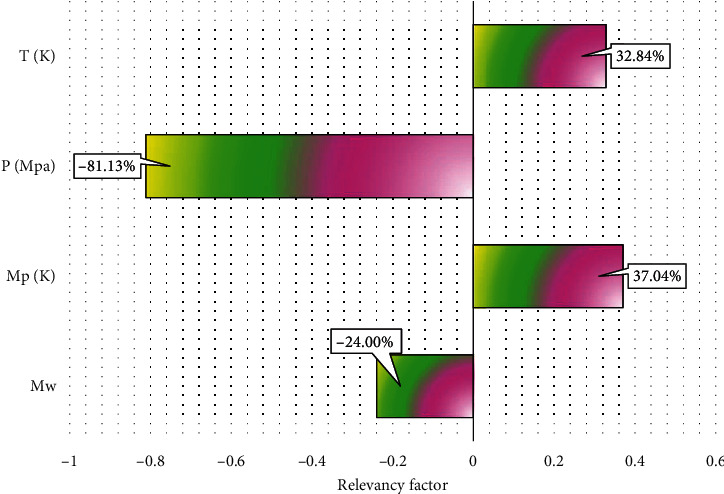
Sensitivity analysis on effective input data.

Then, sensitivity analysis is used to evaluate the dependence of the output values upon input parameters by a relevancy factor (*r*) in the range of +1 to -1 [44, 45]:

(14) $$\begin{array}{c} r=\frac{\sum_{i=1}^{n}\left(x_{K,i}-{\bar{x}}_{k}\right)\left(Y_{i}-\bar{Y}\right)}{\sqrt{\sum_{i=1}^{n}{\left(x_{K,i}-{\bar{x}}_{k}\right)}^{2}\sum_{i=1}^{n}{\left(Y_{i}-\bar{Y}\right)}^{2}}}, \end{array}$$

where *X*_*k*.*i*_ and *Y*_*i*_ are the input and output, as well as $\bar{X_{k}}$ and $\bar{Y}$ are mean values of input and outputs. Here, the higher absolute value of *r* shows the higher impact of arguing variable on the isentropic compressibility. Furthermore, negative and positive *r* values are related to this variable. The effects of the temperature, pressure, molecular weight, and melting point on the isentropic compressibility have been shown in Figure 5. It is notably shown that the temperature and melting point, with *r* values of 32.84% and 37.04%, are the most efficient parameters for the isentropic compressibility determination. Also, the pressure and molecular weight, with *r* values of -81.13% and -24%, are the least efficient variables for the isentropic compressibility, and these variables have a reverse relationship with the output value due to having negative *r* values.

## 5. Conclusion

In this study, we attempted at closing our aim by predicting the isentropic compressibility with the help of various affecting parameters based on a precise and new technique of the ELM. Thus, a comprehensive database is used for training and testing this algorithm. Afterward, the mathematical and graphical modeling was done, and it is shown that this algorithm can predict the isentropic compressibility with high accuracy of *R*^2^ = 1.000, RMSE = 0.0015249, STD = 0.0011567, MRE = 0.19, and MSE = 0.0000023. It is shown that this model has a great ability to learn the behavior of the isentropic compressibility. In addition, it shows a great performance of the testing phase for unknown data points. Also, graphical comparisons demonstrated that the predicted data cover the real data with high accuracy for this algorithm. Last but not least, a comprehensive sensitivity analysis can be used to identify the effects of input variables on the determination of the isentropic compressibility. Temperature and melting point are considered as the most efficient parameters for finding the output values. These findings show that this study can help engineers to simulate and track this parameter in biodiesel. Previous implemented studies need too many parameters which may not be accessible, but our model requires the least number of parameters and predicts the output more precisely.

## Figures and Tables

**Table 1:** Various statistical analyses according to ELM algorithm.

Model	Dataset	*R* ^2^	MRE (%)	MSE	RMSE	STD
Isentropic compressibility (1/Gpa)	Train	1.000	0.18	0.0000020	0.0014306	0.0010710
	Test	1.000	0.21	0.0000032	0.0017773	0.0013781
	Total	1.000	0.19	0.0000023	0.0015249	0.0011567

## Data Availability

The data used to support the findings of this study are provided within the paper.
