# Corrigendum to “Estimation of Isentropic Compressibility of Biodiesel Using ELM Strategy: Application in Biofuel Production Processes”

**DOI:** 10.1155/2022/9760864

**Published:** 2022-05-20

**Authors:** Marischa Elveny, Meysam Hosseini, Tzu-Chia Chen, S. M. Alizadeh

**Affiliations:** ^1^Data Science & Computational Intelligence Research Group, Universitas Sumatera Utara, Medan, Indonesia; ^2^Department of Mathematics, Campus of Bijar, University of Kurdistan, Sanandaj, Kurdistan, Iran; ^3^CAIC, DPU, Bangkok, Thailand; ^4^Petroleum Engineering Department, Australian College of Kuwait, West Mishref, Kuwait

In the article titled “Estimation of Isentropic Compressibility of Biodiesel Using ELM Strategy: Application in Biofuel Production Processes” [1], Adedoyin Isola Lawal was added to the author list in error. With the agreement of all authors, Adedoyin Isola Lawal is now removed from the author list due to the lack of their contribution to the article. The corrected author list is shown above.
